# A Grounded Theory Study on the Intention to Work While Ill among Workers with Musculoskeletal Disorders: An In-Depth Understanding of Workers’ Experiences

**DOI:** 10.3390/ijerph19148700

**Published:** 2022-07-17

**Authors:** Hanizah Mohd Yusoff, Vevya Sundaram, Hanani Nabilah Mohd Sobri, Nor Ba’yah Abdul Kadir

**Affiliations:** 1 Department of Community Health, Faculty of Medicine, Universiti Kebangsaan Malaysia, Kuala Lumpur 56000, Malaysia; drhanie@ppukm.ukm.edu.my (H.M.Y.); hanani.sobri86@gmail.com (H.N.M.S.); 2 Faculty of Social Sciences and Humanities, Universiti Kebangsaan Malaysia, Bangi 43600, Malaysia; aknbayah@ukm.edu.my

**Keywords:** grounded theory, intention, musculoskeletal diseases, workers

## Abstract

Background: Frequent short-term sickness absence is prevalent among workers with musculoskeletal disorders (MSDs). This in return leads to poor productivity in organizations and decreased ability to work among workers. Nevertheless, some workers with MSDs still continue to work despite pain and are able to maintain their productivity. Existing literature on attending work while ill is very limited. Understanding the factors influencing workers’ attendance to work while having symptoms is crucial to help workers live with their MSD productively and healthily. According to literature on behavior theories, the proximal determinant of behavior is one’s intention to engage in that behavior. Thus, this study was conducted to explore the factors that influence the intention to work while ill among workers with MSD. Methods: Twenty-one in-depth interviews were conducted using a semi-structured guide according to a grounded theory approach. Workers with MSD were recruited via a purposive and snowballing sampling until data saturation was attained. Data were analyzed by means of thematic analysis using computer software, ATLAS.ti. Results: Nine major significant themes of factors influencing the intention to work while ill were identified after transcription. From these, a total of six themes were associated with attendance incentives driving workers to attend work while ill (work commitment, work satisfaction, support from colleagues, workplace arrangements, ability to recover at home and ability to manage pain at work) and three themes were linked to attendance requirements (consequences to self, consequences to others and poor acceptance of one’s illness for sickness absence by supervisor and colleagues) faced by workers to attend work while ill. Conclusions: This study underlines the importance of both positive and negative motivators in influencing the intention to work while ill among workers with MSD. Future research suggests comparing both motivators in terms of work performance to aid more workers to work while ill.

## 1. Introduction

Musculoskeletal disorder (MSD) is an eminent public health problem experienced by working populations worldwide [1,2]. MSD consists of injuries that occur in musculoskeletal systems that can cause pain at affected sites, reduction in range of movement, and limitation of function. Work-related MSD (WRMSD) are when the work environment and its execution contribute and worsen the musculoskeletal conditions [3]. Workers report having MSD mostly over the lower back, followed by the shoulders and neck [4,5,6]. MSDs generate the greatest consequences to workers and organizations in terms of occupational losses. One of the losses caused by MSD is the inability to go to work. MSD causes a significant loss of working days [7]. This, as a result, has led to a massive loss of productivity and impacted on economic burdens for employers, organizations, and society in general [8]. MSDs contribute to 0.7% to 1.4% of gross domestic product [9,10].

Workers with MSDs usually take sickness absence for a few days until the symptoms resolved. Although many workers with MSD are confronted with decreased work productivity, the lion’s share of them continue to stay at work and do not report sickness absence. 

Working while having pain symptoms caused by MSD is unique to cases of chronic diseases such as MSD [11]. Past studies have recorded both negative and positive consequences of attending work while ill. From a negative point of view, working with illness can affect one’s work performance and work productivity, hence causing an impact to the quality of work provided [12]. However, working while ill is not always bad. Positive consequences have been associated with attending work while ill. These include high satisfaction and having control over working situations [13]. Identification of factors influencing the intention to work while ill could help in exploring the positive and negative points of view of working while ill among workers with MSD. To date, past studies on sickness absence and work disability have dominated work and health research [11]. Additionally, past studies were bound to overlook this concept by focusing mainly on identifying MSD risk factors, rather than factors that could help workers to work with their MSD in a productive manner. 

The factors influencing the decision to go to work during illness are believed to be prompted by one’s intention or desire to work. Behavioral models can be used to explain the concept of how one’s intention and motivation predicts behavior. Ref. [14] mentioned in his study that one’s motivation to work is important in reaching organizational goals. Furthermore, it must not be forgotten that another model of behavior change is the Theory of Planned Behavior (TPB). This model states that one’s health-related behavior is based on their intention to perform that behavior [15]. One’s behavioral intention is the strongest and most proximal determinant of behavioral performance [16]. Another influential model of work attendance and motivation is the Illness Flexibility Model. This model starts from a condition of a worker experiencing illness that is considered to be absence inducing. This includes chronic illnesses such as MSD, as well as MSD that affects capacity. Capacity is associated with work ability and there are two conditions which determines work ability at illness, namely knowledge and skills. Another aspect of this model is called adjustment latitude. This results in workers who are able to adjust to their illness at work in order to maintain a sufficient work capacity. The main concept is motivation, which decides whether a worker attend work while ill or succumbs to sickness absence. Sources of motivation to work seem to be divided into two different categories: namely, attendance incentives and attendance requirements. Despite major differences between the two categories, they do, however, simultaneously influence workers’ intentions to attend work while ill [17].

Thus, it can be stated that the intention to attend work while experiencing MSD symptoms is a major drive for workers to attend work while ill. Having said that, there is a lack of research examining the factors that distinguish workers who attend work while ill from workers who do not. Workplace environment, motivation and the individual factors integrated into an organization have been demonstrated to be significant and effective in reducing sickness absence among ill workers [18]. Taking into account that our understanding of workers attending work while ill is insufficient, the identification of these factors will help to promote and support workers in working with their MSD in a healthy manner.

## 2. Materials and Methods

### 2.1. Study Design

A qualitative study based on grounded theory was deemed essential for this study since there is a lack of exploration regarding the intention to work while ill. Ref. [19] deduced that a qualitative research design was constructive in exploring new perspectives on workers’ decisions to attend work while ill. This approach allowed incoming new in-depth findings that will be useful for further research and evaluations. This qualitative study applied an in-depth interview approach using a semi-structured interview guide to understand the intention to work among workers with MSD. An in-depth interview was the most suitable method for this study, as it includes sensitive topic related to medical background information and reputation. Hence, potential bias can be reduced by this method compared to focused-group discussions. 

### 2.2. Accessing the Sample

All participants were chosen through purposive sampling and snowballing. Inclusion criteria for this study were workers who were currently employed, aged 18–60, and who had been diagnosed with MSD. To ensure mixture of different perspectives on the intention, we recruited participants with various characteristics according to gender, age, occupation, social background and affected body regions. Twenty-one participants were sampled, and data saturation was attained.

### 2.3. Data Collection

The in-depth interview (IDI) sessions were held between February 2020 and April 2021. The first 4 participants were interviewed face-to-face during their follow-up at an orthopedic clinic. However, due to the Movement Control Order issued by the government related to the COVID-19 pandemic, participants were recruited on the basis of the recommendation of previously recruited participants and by the orthopedic surgeons. Participants were approached by telephone for their consent prior to the interview. Participants who agreed to join were interviewed via Google Meet which is an online video communication platform. All 21 IDI were audio recorded and saved in 2 laptops named by 2 researchers with passwords to open the files.

An interview guide was developed in order to answer the research question. The questions also included the occupational background, MSD background and factors that influence the participants to go to work despite ill (Table 1). Probing questions were also applied in gaining more depth on intention by the participants. In order to make it possible for subjects to speak freely without constraints and to avoid restriction in the mindset of the interviewer, the interview was developed without any explicit theoretical framework.

The interview was conducted in Malay or English languages. Participants were given the flexibility to choose either language and were allowed to have a mixture of both. Field notes were made during and after interview was conducted. They were briefed about the study before the interviews and debriefed at the end of the session. The duration of the interview varied between 45 min to 1 h. All interviews conducted stayed in line with the concept of grounded theory where exploring of the phenomenon and sensitive themes during the interviews process was allowed [20].

### 2.4. Data Analysis

All interviews were transcribed verbatim, and the transcriptions were checked twice against the audio recording. Analysis was performed using ATLAS.ti V.8 Qualitative Data Analysis Software (Scientific Software Development GmBH Berlin, Berlin, Germany). Coding of the transcriptions into meaningful chunks was performed, and the results were compared. The initial analysis of open coding resulted in 65 open codes.

**Figure 1:**
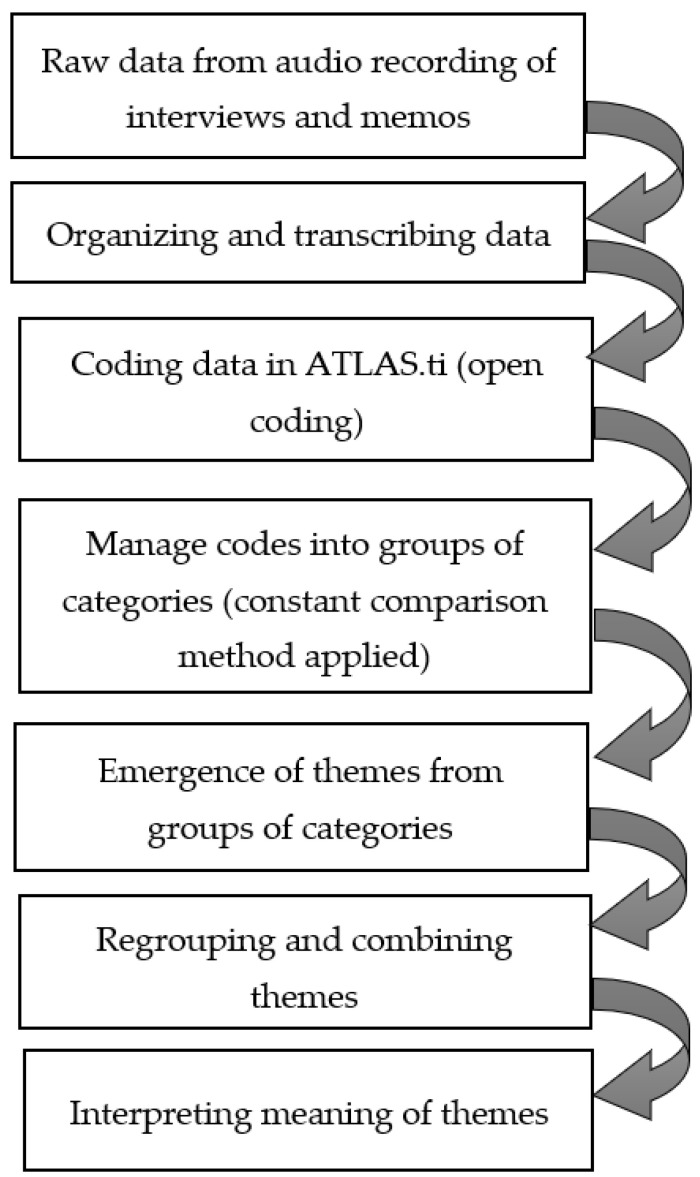
Summary of thematic analysis of data.

In the next analysis phase, categories and themes were identified using the network feature in ATLAS.ti. All open codes and categories were arranged and grouped together to form common themes. All themes were derived from the transcriptions and interviews only, and were not identified at advance. For each process, a memo was written after coding each transcript in order to capture the gist of each interview for the purpose of exploring the participants’ opinion, as opposed to line-by-line coding. This memo also acted as a reflexive note for the thoughts of researcher to minimize potential bias when analyzing the transcripts. Quotes for each theme were translated, organized, and summarized. A summary of the thematic analysis steps is provided in Figure 1.

### 2.5. Data Trustworthiness and Reliability

The reliability of this study is guaranteed by the fact that it followed the principles of grounded theory methods and processes. Data triangulation was performed as a way of increasing the validity and reliability of the study [21]. Purposive sampling ensured external validity, as it was used in the selection of participants was conducted to the greatest extent.

Data accuracy was ensured by reviewing each transcript. Any unclear and vague answers were clarified again with the participants via phone call. Thematic analysis was performed in batches of 4 interviews. As more interviews were carried out, more codes arose to be analyzed. Initially, a total of 18 interviews were performed. However, 3 additional interviews were added to confirm the saturation of the data. Therefore, a total of 21 interviews were finalized and deemed to be saturated for the purposes of this study.

In this study, data triangulation was carried out by 2 experts (Occupational Health Physician and Clinical Health Psychologist) in this subject area to build a coherent justification for the categories and themes built during thematic analysis. The experts challenged the interpretations of the analysts [22]. At the same time, all researchers and experts engaged and discussed coherence and transparency regarding the research process by describing critical elements and themes during data analysis. Finally, all experts agreed with the themes concluded, and stated that the findings were sufficient and had reached the point of saturation. Four important criteria were considered during data triangulation [22]:

(a) What codes and themes were formed during thematic analysis?
(b) What incidents were linked to creating and forming these codes and themes?
(c) How and why were the codes and themes formed?

The criteria above are closely related to grounded theory methods for evaluating the quality of the data obtained [22].

## 3. Results

A total of 21 participants were identified and interviewed. Participants from all three major ethnic groups and various sectors were interviewed. Table 2 represents the sample information of participants interviewed.

**Figure 2:**
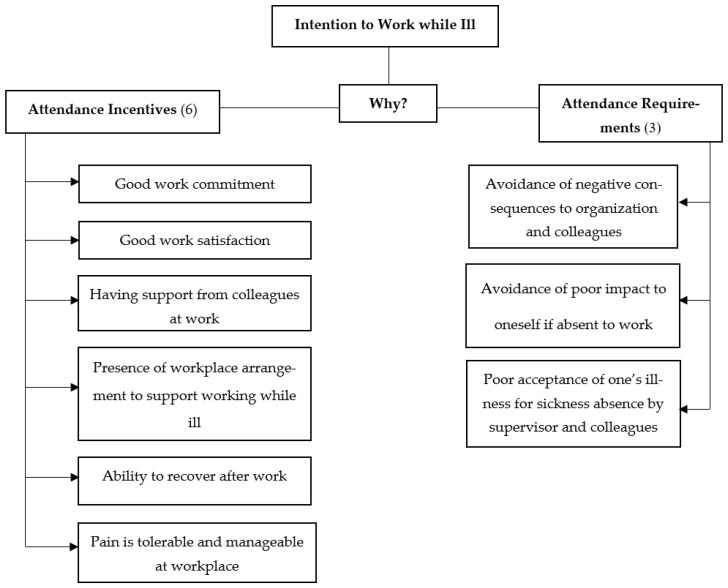
Thematic content of attendance incentives and attendance requirement themes for attending work while ill.

An operational definition was identified for each theme in order for it to be measured and fitted in the context of the themes developed in this study. Seventy-five early codes were compared and identified. After constant comparative analysis, 63 early codes were maintained in order to identify themes. Initially, 15 themes were identified; however, after constant comparison, nine themes were finalized for the study as shown in Figure 2. Of the nine themes, a total of six themes showed positive characteristics of the participants drawing upon aspects of working with MSD, which was decided to be grouped as attendance incentives of workers attending work while ill [17]. Three themes were driven by the requirements faced by workers to attend work while ill to avoid negative consequences to themselves and the organization [17].

### 3.1. Workers Driven by Incentives to Attend Work while Ill

#### 3.1.1. Theme 1: Good Work Commitment

Good work commitment is defined as the intention to work while ill due to feeling responsible towards one’s work. For the majority of participants, a secure income appeared to be a strong motivator to attend work while ill.

PT 1: “*It is good to have motivation although money isn’t everything but you still need money to pay for things? I need it for my children’s expenses and by my medication*”.

PT 7: “*One of the reasons, I still have loans to pay for, so I have no choice but to go. I need to pay for my house loan*”.

PT 13*:* “*Honestly the only motivation is money. I have been jobless for 6 months. What is the motivation? Its money. As long as I can earn good money, you pay me I’ll just be there*”.

Meanwhile, several participants perceived their presence at work to be a necessity to fulfill their responsibility to work despite ill.

PT 15: “*Although I have light duty, due to my 22 years in this profession, I still have to do my job. To make my patients happy, to give what the doctor wants, and my responsibilities. I pity my patients if I cannot help them. So, I have to go*”.

PT 1: “*I feel it is my responsibility. So, yes it is like that. I have to go to work no matter what. As long as I am able to go, I better go. Because I am making a living from my job to eat, something like that. So, have to go work*”.

Being absent without anyone replacing them would mean that the work would not be done. The consequences of this could be very bothering: students would fall behind the education syllabus, deadlines would not be met, and productivity would drop. Most participants had strong feelings of responsibility and kept on working despite being ill.

PT 5: “*I cannot give any excuses. I need to make sure my students examination results are not affected*”.

PT 13: “*I have to go. I need to contribute because my company is paying me my salary. If I don’t go, then my productivity is not good and work projects may not have good quality*”.

#### 3.1.2. Theme 2: Good Work Satisfaction

This theme is defined as the intention to work while ill due to feeling content and happy with one’s daily work. Excellent work satisfaction was shown to be a strong motivator to go to work among the participants. Most notably, simply enjoying one’s job, takes the pain away, was among the points discussed.

PT 6: “*Normally, I talk to my students because no choice, I am teaching language, right? So when I see my students do something awesome, I don’t feel the pain. Although there is pain sometimes, but when I see them, yeah*”.

PT 5: “*Yes, I love my teaching environment. I feel satisfied with my work that I sometimes don’t feel the pain*”.

PT 12: “*I think working in my small little environment there seeing great people I think it just feeds your soul, you feel really good about it. There’s no time to feel tired*”.

Participants expressed feeling of happiness and appreciation when being rewarded and valued by their colleagues and client at work.

PT 15: “*When a patient comes in, and coincidentally the doctor was there, the patient says this and I feel very appreciated and thankful because my patients sees me helping them despite my condition. I take the positive side of this, I feel happy*”.

#### 3.1.3. Theme 3: Having Support from Colleagues at Work

Having support from colleagues was considered highly essential by the participants in terms of their intention to stay at work despite being ill. The idea of receiving emotional support and tremendous teamwork from colleagues and subordinates evidently increased participants’ intentions to go to work despite being ill. This theme is defined as the intention to work while ill due to the support received at workplace.

PT 6: “*I would say it is my environment, my colleagues at work are so understanding*”.

PT 14: “*I quite enjoy for this work and because of the environment, quite harmony, we helped up each other. We backed up each other when necessary. Yeah, then work together. I think that environment is quite important for me. And in our bank it is very harmony, and especially in our team there’s teamwork there*”.

Participants also expressed how they were able to delegate their tasks to their colleagues when they are not capable to do it themselves due to their illness. Amazing teamwork can definitely be concluded from this and motivates ill participants to continue working despite ill.

PT 11: “*I normally assign tasks to my colleagues. They will help me*”.

PT 13*:* “*My colleague will help me. Because I will focus on my walking and the kitchen. I won’t directly handle the customers. She will be collecting orders, packing. She moves a lot. I would move less and just focus on the cooking*”.

#### 3.1.4. Theme 4: Presence of Workplace Arrangements to Support Working While Ill

This theme is defined as the intention to work while ill due to the presence of workplace arrangements to support working while ill. A large majority of participants mentioned how work arrangements contributed as a powerful success factor to attend work despite being ill. They described the benefits of having an understanding and flexible organization who made work arrangements unofficially in order to support them working while ill. Examples given included the privilege to turn up late or take time off without facing many inquiries from superiors, the arrangement of light duties, financial cover, and facilities in the workplace for resting and relieving pain, while some were able to change their workplace in order to avoid painful work tasks. Participants emphasized the importance of a good working relationship with their superiors and being given resting periods while working. Several participants felt appreciated by being given light duty by their superior.

PT 3: “*At my office here, there is a physiotherapy clinic. Therefore, staffs here can attend the clinic with an appointment for free of charge and get any advice from there. Besides, there is a place like a capsule where I can rest if I am tired or need to rest my back if I am in pain*”.

PT 7: “*My management gave me the privilege to work in the lower floor because I am unable to climb to the higher floor. He gave me the privilege because I am ill*”.

PT 15: “*After a while, the specialist doctor gave me light duty 4 times. But I did not take it because I am still capable to work. After the fourth time, after insisting I take light duty, I took it. It has been 2 years*”.

The presence of workplace arrangements could contribute to high intentions among these participants to work while ill. The role of supervisors in organizations in designing these arrangements may facilitate more workers to attend work while ill.

#### 3.1.5. Theme 5: Ability to Recover after Work

This theme is defined as the intention to work while ill because one has the potential to recover at home after work. This recovery is attained by the ability to avoid carrying out non-work responsibilities or delegate these responsibilities to family members or helpers after returning home from work to rest and recover from pain felt.

PT 2: “*Sometimes, I do not do much work when I am home. I have a helper, she comes every evening. If I am unable to do house chores, she helps me*”.

PT 4: “*My kids help me. That is fine. It is not that I will be stressed when I come home. Because they help with things at home*”.

Ability to rest and recover at home after a long day at work definitely boost’s ones intention to go to work despite being ill. When participants shared that that were able to rest, it meant they were able to recharge themselves before facing another busy day at work the next day. The idea that they can recuperate at home enhance their intention to go to work despite having the illness.

PT 17: “*When I go home, I can rest completely. I take a shower, do my prayer and I will completely rest, lying down*”.

#### 3.1.6. Theme 6: Pain Is Tolerable and Manageable in the Workplace

One of the themes that was found to motivate participants to attend work while ill was the participants ability to tolerate and control pain while at work.

PT 7: “*Normally I would use a muscle sprain spray or take a painkiller or sometimes I bring my ointment with me. Once I feel ok, I would resume teaching*”.

PT20: “*If I am feeling pain, I will take a Paracetamol and resume work as usual. Not a problem*”.

Some participants also described that their pain was not as severe and they did not see their pain as a hindrance to their work performance.

PT 3: “*Yes, like I said, my job scope does not involve physical activities, only doing reports. So it is not a problem, as I can go, I go*”.

### 3.2. Workers Driven by Requirements to Attend Work While Ill

#### 3.2.1. Theme 7: Avoidance of Negative Consequences to Organization and Colleagues

This theme is defined as the intention to work while ill to avoid negative consequences being suffered by their colleagues and their organization. Ill workers are often concerned that they may be a burden to their colleagues and organization and worry what people would think of them. To avoid being criticized, they would have no choice but to attend work while ill.

PT 1: “*I wouldn’t want to say my workplace is not best. It’s like when I’m on sick leave, they criticize me. Because here, they have Key Performance Indicator, before COVID when there’s KPI, I can’t punch card in for sick leave for more than 3 days. So any leave, emergency leave is included in normal sick leave. My colleagues also say I can’t take MC for too long or it will affect my KPI*”.

PT 2: “*I pity my colleagues. Then, they have to work double shifts to cover for me. From seven hours, she has to work nine hours because of me*”.

PT 18: “*My manager will have a headache trying to arrange a replacement for me*”.

#### 3.2.2. Theme 8: Avoidance of Poor Impact to Oneself if Absent to Work

This theme is defined as the intention to work to avoid negative consequences of absence to oneself. Not every organization ensures job security for their ill workers. Participants raised concerns regarding their job security, whereby they were concerned that they would be fired or get transferred to another department if they took prolonged sick leave due to their illness.

PT 2: “*I don’t feel that great maybe. Maybe I might be transferred to another department in the workplace if I don’t perform well at work*”.

PT 9: “*I was thinking if I am on a long sick leave, I’m afraid of being let go. I am just an employee*”.

Participants shared the consequence to themselves in cases where they needed to cover accumulated work if they did not go to work despite being ill.

PT 4: “*If I am absent on that day, I will have an added responsibility, additional workload to do. If I don’t finish the syllabus, I have to conduct extra classes and it takes extra time to teach and finish it. So, it’s better if I just work as per usual to avoid that*”.

#### 3.2.3. Theme 9: Poor Acceptance of One’s Illness for Sickness Absence by Supervisor and Colleagues

Supervisor and colleagues undermining one’s illness is another important factor that drives workers to still continue working although they are ill. This theme is defined as the intention to work while ill due to poor acceptance of one’s illness to take sick leave by supervisor and colleagues. Participants expressed fear because their superior interpreted their illness as something not serious and being afraid if their work merit points will be taken away if they are nor present to work.

PT 1: “*I have to accept my superior’s orders because I work under his supervision right. I cannot complain. Once when I told I was in pain, my superior said I look fine and I can still do my work*”.

On the other side, participants also shared that the illness they were had experienced was common among their colleagues in the organization. Therefore, if their colleagues were able to work while ill, so could they.

PT 2: “*I think it is normal. When we talk about slip disc, there are others here who has slip disc too. Because we are in airline, there’s ground handling*”.

PT 4: “*There are so many teachers having knee pain, so many. Knee pain is normal here. If I feel pain, I bet others feel it too. So, it is best I don’t mention my pain because everyone has it too”.*

Participants also shared that their supervisors and colleagues undermined the legitimacy of their absence due to sickness. This is particularly difficult to some, especially for those who work in the healthcare and teaching fields, as it is not convenient to find a replacement easily. This is because each worker in the organization already has a scheduled working shift.

PT 2: “*So, I want to change my schedule, they would be like why should I change it? It is difficult for me*”.

PT 7: “*To replace me, there are a few teachers who can replace, but not at all. Other teachers have their own subjects and materials to teach*”.

Therefore, these participants had to go to work despite being ill. These are among the reasons that causes participants to still attend work despite being ill.

## 4. Discussion

This current study provided a deep understanding and a unique perspective on the intention to work while ill. It can be significantly observed that there are several factors that emphasizes a worker’s intention to work while ill.

One of the main findings discovered is that the intention to work was seen to be influenced by factors that distinguished the incentives and requirements to attend work while ill. It was observed that these two groups of factors operate simultaneously in influencing MSD workers’ intention to attend work when ill. This study also demonstrates the receptivity of colleagues and superiors in an organization and the importance of these factors towards ill workers’ intention to attend work. 

It can be seen that in enhancing workers’ intentions to work while ill, supervisors and colleagues in a workplace play and influential role. These findings suggest that the undermining of one’s illness by supervisors enhances participants’ intention to go to work while ill. When the supervisor’s attitude towards the participants is not supportive, this induces pressure on the participants to attend work while ill. The findings from [23] reported similar findings, whereby employees with low supervisor support had a higher return-to-work rate. However, this is contrary to evidence from previous studies, which suggested that low supervisor support leads to a longer duration of work disability [24,25]. A meta-ethnography study introduced two themes, including not being believed and not being judged, which showed an influence on work relationships and returning to work. A lack of employer understanding of chronic pain could have a negative impact on someone’s ability to return to work [26]. Low social support from supervisors might enhance the feeling of job insecurity, because it may be perceived as having a bad relationship with the supervisor [23]. Participants voiced concerns that their colleagues and supervisors would undermine their sickness absence if they were unable to attend work due to illness. In this case, superiors must play a role in making sickness absence among ill workers convenient without undermining them. According to [27], in Malaysia, for an employee to be entitled a sick leave, they must first be examined by a registered medical practitioner and be certified as being ill enough to require a period of sick leave. To take sick leave without permission or being examined is misconduct that could potentially justify termination of employment. Therefore, supervisors should ensure they have a proper system for recording and monitoring employees’ sick leave. This may, in return, ease workers in taking sick leave if they are incapable of attending work due to their illness. If the human resources department does not follow up on employees with request for their medical certificate, this can cultivate an unhealthy culture among employees. This would result in employees being forced to attend work despite being ill, as well as being tempted to test their limits.

On the theme of presence of workplace arrangements to support working while ill, participants have shared how their supervisors and organizations provided them with adjustments such as physiotherapy facilities and light duties. Furthermore, having a good relationship with the necessary support for making adjustments to work schedule, such as time-offs, was also reported. Ref. [28] emphasized in his study how workplace adjustments facilitate the successful return to work among workers with chronic pain. He also mentioned in his study the importance of a good working relationship with managers, and how helpful this is to workers with chronic pain. Another study by [29] identified that rehabilitation centers may contribute as a facilitator among patients with low back pain. 

In enhancing the intention to work while ill, participants themselves play a major role. By having a strong work commitment and good degree of work satisfaction, participants felt more motivated and pushed to go to work despite being ill. Participants’ feelings of responsibility and happiness towards their work would not feel like a burden to them. A meta-analysis by [30] emphasized on the “want to” rather than the “have to” aspect of behavior is implicated by the impact of job attitude and organizational justice. Being affectively committed to one’s organization, liking one’s job, and being engaged in work motivates good attendance, even in the face of medical discomfort. Each of these positive affective states has been shown to strengthen and cultivate organizational citizenship behavior [31]. Ref. [32] described how one’s intentions and passion towards one’s work enhance organizational commitment. At the same time, Ref. [33] also pressed how workers who show loyalty and readiness to face challenges at work exhibit a good indicator of their work commitment, leading to good organizational behavior.

With respect to the above, in the current study, participants did mention about the consequences upon themselves if they took sick leave due to illness. Participants were afraid of being let go by their organization due to sickness absence. Ref. [30] suggested that employees with job insecurity were more likely to work despite feeling ill, with the aim of securing their jobs. Ref. [34] shared similar findings by reporting that insecure workers might choose to work while sick rather than to take sick leave because they are afraid of dismissal.

Obtaining support from colleagues is definitely seen as being a major boost in intention among ill participants in terms of attending work while ill. In this study, most participants were afraid to burden their colleagues with extra work if they were absent. Managing responsibilities between colleagues may seem challenging, as it may cause unequal work distribution when an ill worker is absent or in pain, which may lead to injustice [35]. In this case, participants have no choice but to attend work in order to reduce the tension and burden felt by their colleagues. However, by contrast, if colleagues shower ill participants with support and understanding, this may in turn also motivate participants to be present at work. Some participants voiced having supportive colleagues who were always ready to help distract them from thinking about their pain [36]. Leaders and colleagues working with employees with a supportive capacity was an important factor in motivating employees to stimulate their job crafting abilities [37,38]. However, while analyzing these findings, this theme appeared to be paradoxical. On the one hand, participants were motivated to work because their colleagues were supportive of them and their illness, making it easier for them to work while ill. On the other hand, some colleagues were always available to lend a helping hand, causing them to question whether or not to attend work while ill. This is because they know their colleagues would be considerate regarding their illness and would do their work for them, thus constituting a paradoxical tension. This paradox was not explored further, as the research question only highlighted the reasons motivating workers with MSDs to attend work while ill and not otherwise. A study by [39] noted that paradoxes seems to be a trend in organizational studies in explaining conflicting demands and opposing perspectives.

Based on these findings, it can be seen that the ability to rest and recover at home after a long day at work precedes one’s intention to attend work while ill. From a psychological point of view, it is when people feel capable and ready to continue with the current demands or to meet new demands. In other words, a common expression referring to this effect is “charging the batteries”, work and rest need to alternate and consists a “cycle of work and rest”. In short, when taking rest to recover at home, it reliefs the demands experienced at work. This relief will allow to replenish the resources that have been used and able to go to work the next day with a fresh start [40].

In this study, the majority participants did not see their pain as a barrier for them to work when ill. They were able to find methods to control their pain while ill. These findings are in opposition to most studies, which state that pain is associated with a reduction in the ability to work, work productivity, and an increase in psychological distress [41,42]. To explain the findings from this study, it can be said that participants may still choose to go to work despite experiencing pain because they are afraid that they might burden their colleagues and superiors. It still comes down to the view of colleagues and superiors. This explanation is also discussed in another study, which reported that workers with chronic pain were concerned that they may be a burden on their colleagues and worried what others might think of them [28]. Another study by [43] reported that when participants started to believe there were ways to control their pain, they would reconstruct their representation to include in the term “new normal pain”. This “normal pain” is sustained among workers in order to think that they should be able to bear the pain, without worrying or taking any precautions at work.

The Illness Flexibility Model approach played a role in during developing the codes and categories. This process of coding is in keeping with the recommendation that the use of existing theory has to earn its way into analysis [20,44]. Staying within the concept of the study, the workers’ motivation to act in the Illness Flexibility Model can have two different origins. The first includes conditions that drive workers to want to attend work despite illness. These are called attendance incentives, where, in other words, workers consider they have certain qualities at work such as a supportive and friendly climate at work, plus those who simply enjoy and feel content with their job. The second origin of the motivation to work while ill are attendance requirements. In this case, however, workers are supposed to act in a way to what they perceive they ought to do. Workers are bound to adhere to perceived environmental conditions as to what the other party wishes and needs. Non-adherence to these conditions may lead to negative consequences which can affect the other party such as their supervisor or workmates. Therefore, workers have no choice but to adhere to meet these requirements and attend work while ill. These two distinctive group of factors, has been implied in the means of this study to differentiate two groups of workers and why they choose to attend work while ill [17,45].

This study provided a range of factors affecting a workers’ intention to work while ill. However, the role of compensation policy and social security system should be taken into account in finding an explanation on the factors influencing the intention to work while ill [46]. Studies by [47] suggest environmental factors such as job characteristics and the stakeholders involved are among other important explanatory variables explaining and determining working while ill among workers. Cultural values have also been shown to influence work attitudes of employees such as work commitment and job satisfaction respectively, which supports the findings of the study. However, ref. [48] discovered that familism, ethnicity, traditional leadership, religious beliefs, and fear of the unknown, as well as important cultural beliefs, have consequences for management practices in a workplace. Moreover, Asian culture and western culture differ in the working world. Asian workers are more influenced by work-related and societal culture, and are therefore prone to working while ill, which may in turn cause long-term health issues. Therefore, significant employee-related and culturally relevant research is needed in the future in order to be able to achieve an optimal working environment, in which the limited resources are allocated to achieving maximum productivity [49,50].

Findings such as ability to recover after work, good work commitment, and work satisfaction can be applied to the benefits of workers with MSD. Work, in general, is good for one’s well-being, as it provides many benefits, such as income and participation in society, while also being central to individual identity and social roles. Moreover, several incentive factors, such as presence of workplace arrangements, can be provided by employers in order to support workers with MSD working while ill in a healthy manner. Good communication between employer and workers can be enhanced, thus avoiding a negative perception of worker illness and avoiding negative consequences towards both them and the organization. Work is considered an important part of life. Therefore, a shared responsibility between the employer and ill workers is needed, in order to effectively support working while ill among workers with MSD. 

## 5. Strengths and Limitations

To the best of our knowledge, this study is among the first qualitative studies investigating the intention to work among workers with MSD. This approach provides an opportunity to capture a range of views regarding workers’ intentions to go to work despite having an illness. The findings of this study can be used by employers to understand the intention of workers to attend work while suffering from MSD. However, due to the COVID-19 pandemic, the IDIs performed in this study were carried out mostly via a virtual platform, which may limit the exploration of the views and non-verbal signs of the participants. 

## 6. Conclusions

The results of this study suggest that there are nine themes that can be extracted from the intention to work while ill among MSD workers. The intention to work was seen to be driven by incentives and adherence of workers to attendance requirements. Future studies may look into work productivity among those who work because there are incentive to work while ill compared to those who need to work while ill due to the attendance requirements. This study also provided the themes that can be used in the development of a validated questionnaire to measure the intention to work despite being ill that can be used in future studies.

## Figures and Tables

**Table 1:** Interview guide to be used in data collection.

Phase	Phase	Question (s)	Possible Probing Questions
Consent and demographic data	Consent	Name, age, educational background, occupation, audio recording permission	-
Introductory	Occupational background	What do you work as? What is your main role in the workplace?	-
Focused topic	Intention to work while Ill	Why do you still go to work despite being ill?	Would you explain that? Why do you say so? Please give an example? What modification can be done to solve the problem?

**Table 2:** Demographic characteristics of participants (*n* = 21).

Characteristics	Value *n* = 21 (%)
**Age (years)**	
<30	3 (14.29)
30–39	9 (42.86)
40–49	5 (23.81)
≥50	4 (19.05)
**Ethnic**	
Malay	16 (76.19)
Chinese	4 (19.05)
Indian	1 (4.76)
**Gender**	
Male	8 (38.10)
Female	13 (61.90)
**Employment Sector**	
Healthcare	4 (19.05)
Education	4 (19.05)
Manufacturing	7 (33.33)
Other Services	6 (28.57)
**Region of musculoskeletal pain**	
Upper Limb	6 (28.57)
Axial	9 (42.86)
Lower Limb	6 (28.57)
